# Comprehensive genetic testing combined with citizen science reveals a recently characterized ancient *MC1R* mutation associated with partial recessive red phenotypes in dog

**DOI:** 10.1186/s40575-020-00095-7

**Published:** 2020-11-05

**Authors:** Heidi Anderson, Leena Honkanen, Päivi Ruotanen, Julia Mathlin, Jonas Donner

**Affiliations:** Wisdom Health, Helsinki, Finland

**Keywords:** Ancient, Canine, Dog, Coat color, Domino, Grizzle, Pheomelanin, Eumelanin, MC1R, Reduced-function variant

## Abstract

**Background:**

The Melanocortin 1 Receptor (MC1R) plays a central role in regulation of coat color determination in various species and is commonly referred to as the “E (extension) Locus”. Allelic variation of the *MC1R* gene is associated with coat color phenotypes *E*^*M*^ (melanistic mask), *E*^*G*^ (grizzle/domino) and *e*^*1–3*^ (recessive red) in dogs. In addition, a previous study of archeological dog specimens over 10,000 years of age identified a variant p.R301C in the *MC1R* gene that may have influenced coat color of early dogs.

**Results:**

Commercial genotyping of 11,750 dog samples showed the R301C variant of the *MC1R* gene was present in 35 breeds or breed varieties, at an allele frequency of 1.5% in the tested population. We detected no linkage disequilibrium between R301C and other tested alleles of the E locus. Based on current convention we propose that R301C should be considered a novel allele of the E locus, which we have termed *e*^*A*^ for “e ancient red”. Phenotype analysis of owner-provided dog pictures reveals that the *e*^*A*^ allele has an impact on coat color and is recessive to wild type *E* and dominant to the *e* alleles. In dominant black (*K*^*B*^/*) dogs it can prevent the phenotypic expression of the K locus, and the expressed coat color is solely determined by the A locus. In the absence of dominant black, *e*^*A*^/*e*^*A*^ and *e*^*A*^/*e* genotypes result in the coat color patterns referred to in their respective breed communities as domino in Alaskan Malamute and other Spitz breeds, grizzle in Chihuahua, and pied in Beagle.

**Conclusions:**

This study demonstrates a large genotype screening effort to identify the frequency and distribution of the *MC1R* R301C variant, one of the earliest mutations captured by canine domestication, and citizen science empowered characterization of its impact on coat color.

**Supplementary Information:**

The online version contains supplementary material available at 10.1186/s40575-020-00095-7.

## Background

Coat color in dogs is determined by expression of two melanin pigments, eumelanin (black/brown) and pheomelanin (yellow/red) and by spatial and temporal regulation of these pigments’ expression in the body and in the individual hair shaft. *Melanocortin 1 Receptor* (*MC1R*), known as the E locus, represents the key signaling molecule on melanocytes inducing expression of enzymes responsible for eumelanin synthesis. The alleles in order of dominance at the E locus are: *E*^*M*^ (melanistic mask) > *E*^*G*^ (grizzle/domino) > *E* (wild type) > *e*^*1–3*^ (recessive red) [1–5]. The *e*^*1–3*^ variants result in loss of gene function and consequently for dogs with the genotype *e*/*e* only pheomelanin (yellow/red) pigment is present. The allelic variant *e*^*1*^ is common and found in a wide variety of dog breeds [4, 6], while the *e*^*2*^ and *e*^*3*^ alleles represent rare additional *e* variants found in Australian Cattle Dogs and white Alaskan and Siberian Huskies [1], respectively. The *E*^*G*^ allele is one of the rarest trait-associated alleles present in dogs, requiring specific genotype combinations at more than one locus in order to produce a domino or grizzle phenotype. This phenotype has been characterized in Afghan Hounds and Salukis only [3, 6]. *Agouti Signaling Protein* (*ASIP*) gene, known as the A locus, is an inverse agonist of MC1R inhibiting eumelanogenesis and promoting pheomelanogenesis. Four known alleles in order of dominance are *a*^*y*^ (fawn) > *a*^*w*^ (wolf sable) > *a*^*t*^ (tan point) > *a* (recessive black) [7–9]; of these *a*^*w*^ is considered the wild type *ASIP* allele. The most dominant allele *a*^*y*^ represents a gain-of-function mutation causing an increased expression of pheomelanin, whereas the alleles recessive to wild type show reduced-function and increased expression of eumelanin [8]. There is variable expression of dark hairs in *a*^*y*^ fawn dogs; when no dark hairs are present the *a*^*y*^ fawn appears similar to recessive red, while abundant display of dark hairs produces an *a*^*y*^ phenotype highly similar to the wild type. Such variable expression of ASIP expression and phenotype could be due to epigenetic mechanisms as shown in mice [10, 11]. In addition, combinations of more than two allelic variants at the A locus were identified, with variable phenotypic manifestation, in a small number of dog breeds [12]. The *canine beta-Defensin 103* (*CBD103*) gene, known as the K locus, is a neutral antagonist of *MC1R*. The dominant *K*^*B*^ (dominant black) allele prevents ASIP inhibition enabling high levels of basal receptor activity resulting in solid eumelanin coat color. Similarly, a phenotypically intermediate *k*^*br*^ allele (brindle) of the K locus produces dominantly overlaying eumelanin stripes on A locus determined background [13, 14]. In the presence of *k*^*y*^ (wild type) allele of the K locus, the A locus is expressed normally. The phenotypic expression of the A and K loci is dependent on the presence of at least one functional *MC1R* allele, and are not expressed in the presence of an *e*/*e* genotype. Coat color variation in dogs and various other domesticated species is a result of domestication and intentional selection of novel phenotypes by humans and neither is explained by relaxation of natural selection [15].

Coat color variation represent one of the first effects of domestication, where the selection for novel non-camouflage coat colors may have helped identification and tracking of animals in husbandry, differentiation of domesticated animals from wild herds, or be explained by human attraction for novelty [16]. Experiments have also shown that selection for animal behavior, especially tameness, also results in alterations in physical traits including color [17]. In dogs the R301C variant of *MC1R* and the dominant black *K*^*B*^ allele of the *CBD103* gene were both found in the DNA of over 10,000 years old Siberian and South-Eastern European dogs [18]. Only dog-like samples and no wolf-like samples were found to carry R301C or the *K*^*B*^ allele suggesting that these variants could represent some of the first coat color variants present at the time of early dog domestication [18]. While the R301C variant has been found in two modern day breeds the Alaskan Malamute and the Siberian Husky, its phenotypic impact could not be determined [18]. Potential reduced-function was postulated based on functional characterization of a mutation at the same codon position (301) together with two other polymorphisms found in 43,000 years old woolly mammoth that resulted in nearly complete loss of basal activity and ~ 65% reduction in efficacy to agonists *alpha*-Melanocyte Stimulating Hormone (α-MSH) [19].

The aim of this study was to utilize the potential of commercial genetic panel screening to genotype large numbers of dogs for the presence or absence of the R301C variant of *MC1R* found in prehistoric dogs to better understand its frequency and distribution in modern dog breeds. To further unravel the potential influence of R301C, coat color variant genotypes were correlated with the dog’s actual coat color phenotype from photos provided by dog owners.

## Results

### Ancient R301C variant of the *MC1R* gene is present in various breeds of today

**Fig. 1:**
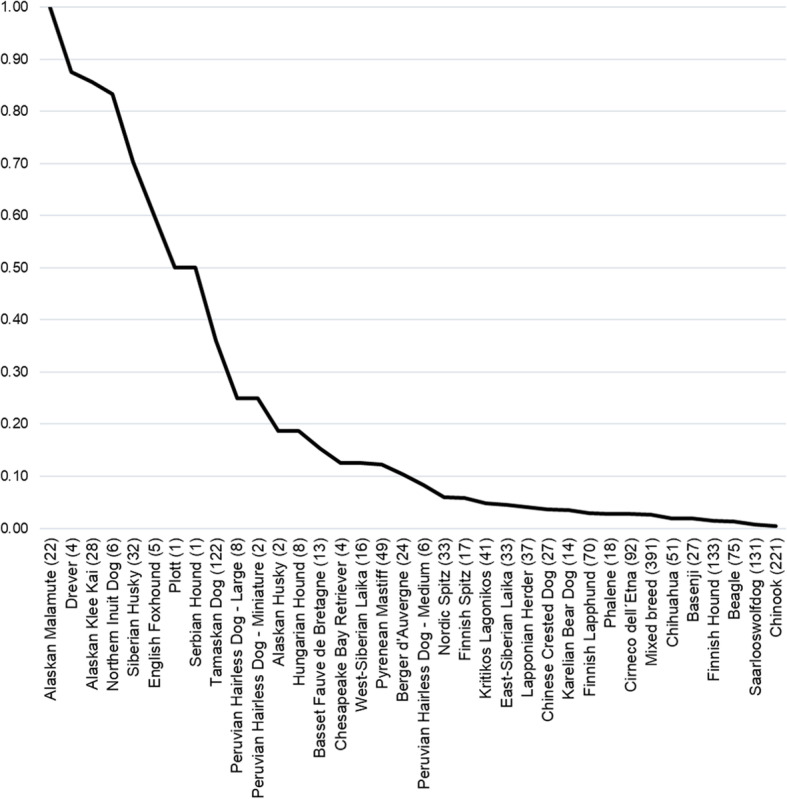
Allele frequency of the R301C variant in 35 breeds and breed varieties. The black line indicates the allele frequency of R301C in each breed or breed variety. The number of dogs in each breed analyzed for the presence of the R301C variant is indicated after the breed label

To screen for the presence and frequency of the ancient R301C variant of *MC1R* in today’s canine population, 11,750 dog samples were genotyped as a part of a custom-designed microarray panel test commercially available as MyDogDNA™/Optimal Selection™ Canine Genetic Breeding Analysis. The R301C variant was present in a total of 265 tested dogs representing 35 different breeds and breed varieties as well as mixed breed dogs. The allele frequency for R301C in all dogs representing 304 different breeds and mixed breeds was 1.5% (*N* = 11,750; Fig. 1 and Table S1). The R301C frequency in the tested Alaskan Malamute population was 100%. The additional 34 breeds in which the R301C variant was found could be classified into old Nordic Spitzes (East-Siberian Laika, Finnish Lapphund, Finnish Spitz, Karelian Bear Dog, Lapponian Herder, Nordic Spitz, Siberian Husky, West-Siberian Laika), other Primitive Spitz Type dogs (Basenji, Cirneco Dell’Etna, Kritikos Lagonikos, Peruvian Hairless Dog – Large, Medium and Miniature), Scent Hounds (Basset Fauve de Bretagne, Beagle, Drever, English Foxhound, Finnish Hound, Hungarian Hound, Plott, Serbian Hound), one gundog breed (Chesapeake Bay Retriever), one guardian dog breed (Pyrenean Mastiff), three Companion and Toy Dog breeds (Chihuahua, Chinese Crested Dog, Phalene),some recently created breeds (Alaskan Husky, Alaskan Klee Kai, Chinook, Northern Inuit, Tamaskan Dog, Saarlooswolfdog), and a nearly extinct sheepdog of the Auvergne region in France (Berger d’Auvergne). In this study sample the R301C variant was not found in dog breeds with Eastern Asian origin (Akita, Chow Chow, Hokkaido, Kai, Kishu, Shar Pei, Shiba, Shikoku, Korean Jindo Dog) or Middle Eastern/Central Asian origin (Afghan Hound, Saluki, Tibetan Mastiff, Tibetan Spaniel, Tibetan Terrier, Lhasa Apso, Shih-Tzu, Central Asian Ovcharka).

### R301C is a novel alternative allele of the E locus

To elucidate the relationship of R301C and other known E locus variants, genotypes were obtained for *E*^*M*^ (melanistic mask), *E*^*G*^ (grizzle/domino) and *e*^*1*^ (recessive red) alleles of the *MC1R* gene. Two rare additional recently characterized *e* allelic variants [1]; *e*^*2*^ discovered in Australian Cattle Dog and *e*^*3*^ discovered in Siberian Husky were not genotyped as a part of this study. The R301C variant and the tested E locus variants showed no linkage disequilibrium in the 262 dogs with diverse breed background in which it was found present. The R301C variant was not present in dogs with two copies of the tested E locus variants; *E*^*M*^, *E*^*G*^ or *e*^*1*^, while in dogs with two copies of the R301C variant no *E*^*M*^, *E*^*G*^ or *e*^*1*^ variants were present. Also, no more than one copy of *E*^*M*^ or *e*^*1*^ variants was present when one copy of R301C was found. The rarest *MC1R* coat color variant, the *E*^*G*^ allele, is only found in one of the dog breeds, Kritikos Lagonikos, in which R301C was identified. However, in this study sample no individuals carrying both *E*^*G*^ and R301C variants were identified.

Notably, using current conventional practices for calling of E locus genotypes at commercial genotyping laboratories, dogs carrying R301C would have been interpreted as carrying *E*. As our findings suggested that R301C rather represents an independent alternative allele at the E locus, we refer to it as *e*^*A*^ (for ancient red *e*) for clarity hereafter.

### *e*^*A*^ allele of *MC1R* explains presence of domino patterning in Tamaskan dog

**Fig. 2:**
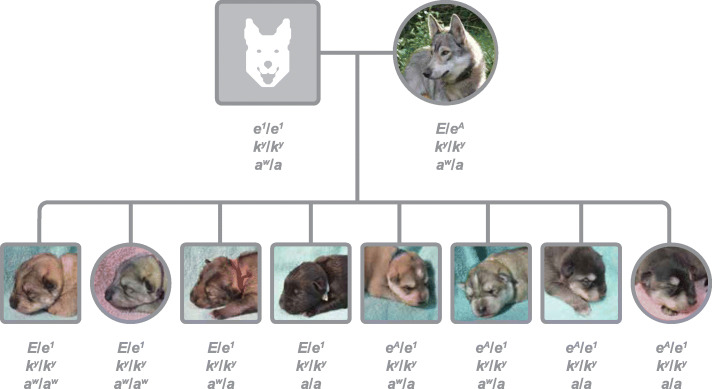
The *e*^*A*^ allele associated domino pattern is recessive to the wild type *E* allele. Mating of recessive red (cream colored) *e*^*1*^*/e*^*1*^ sire to *E/e*^*A*^ wolf sable dam in the Tamaskan Dog breed, resulting in four normal wolf sable puppies with *E*/*e*^*1*^ genotype and four puppies with domino pattern, of which two express domino on wolf sable and two express domino on recessive black. In newborns, domino pattern is visible as large pheomelanin colored areas on both sides of the muzzle, pheomelanin areas around the eyes and overall as a lighter coat color of the body with dark bar of hair remaining on the back side around the vertebra

**Fig. 3:**
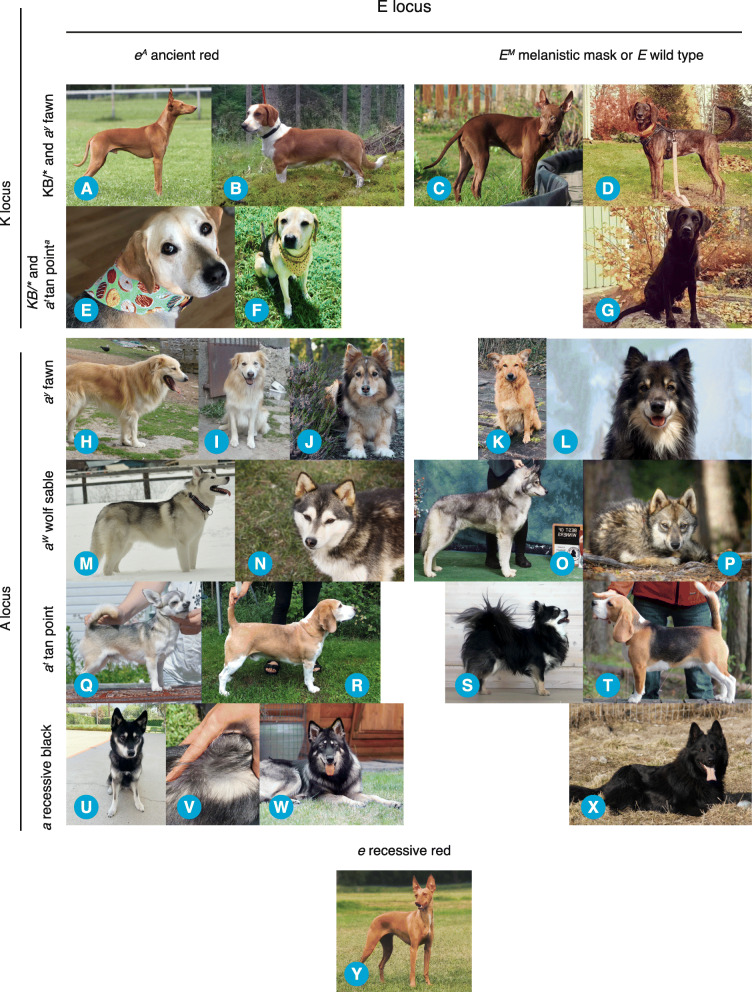
Photos representing the phenotypic impact of the *e*^*A*^ allele. The *e*^*A*^/*e*^*A*^ and *e*^*A*^*/e*^*1*^ genotypes mask the presence of *K*^*B*^ (or *k*^*br*^) at the K locus to produce phenotypes representing the dogs A locus genotype; *a*^*y*^ fawn in Cirneco dell’Etna (**a**) and in Drever (**b**); differing from the rare *K*^*B*^ solid eumelanin shade in Cirneco dell’Etna (in which the eumelanin shade is brown due to variants in the *TYRP1* gene) (**c**); or *k*^*br*^ brindle patterning displayed in Plott (**d**); a saddle tan modified *a*^*t*^ tan point in mixed breed dog (**e**-**f**) instead of solid dominant black in Labrador Retriever (**g**). The *e*^*A*^ phenotype in dogs expressing patterns of the A locus *a*^*y*^ fawn; is cream in Berger d’Auvergne (**h**-**i**) and domino in Finnish Lapphund (**j**) compared to fawn in Berger d’Auvergne (**k**) and heavily shaded fawn in Finnish Lapphund (**l**); *a*^*w*^ wolf sable also presents as color pattern domino in Siberian Husky (**m**) and in a mixed breed dog (**n**), compared to typical wolf sable in Siberian Husky (**o**) and in mixed breed dog (**p**); similarly *a*^*t*^ tan point shows a color pattern called “grizzle” in Chihuahua (**q**) or reduced saddle tan patterning called “pied” in Beagle (**r**); (**j**) compared to normal tan points in Chihuahua (**s**) or typical saddle tan patterning in Beagle (**t**); a recessive black dog manifests tan points with pale hair root in Alaskan Klee Kai (**u**-**v**) or wolf sable in Tamaskan Dog (**w**) instead of uniform recessive black in German Shepherd Dog (**x**). Taken together, ancient red *e*^*A*^ phenotypes manifest as a wide variety of *partial* recessive red coat colors expressing less eumelanin compared to dogs with wild type *E*, while no eumelanin is expressed in *e* recessive red exemplified by a Cirneco dell’Etna (**y**)

To interpret the phenotypic impact of the R301C variant, *e*^*A*^, on the dog’s coat color, also genotypes for *Canine Beta-Defensin 103* (*CBD103*) and *Agouti Signaling Protein* (*ASIP*) were obtained for the analysis. Color phenotypes were available for 125 (47%) dogs of the 265 dogs identified with one or two copies of the *e*^*A*^ allele in this study. Phenotyping using dog owner-provided photos initially focused on the Tamaskan Dog breed, which represented 35% (*N* = 43) of the dogs with phenotype information available. These dogs were carefully examined to elucidate the potential phenotypic effect of *e*^*A*^ within a single breed. The expected coat colors for these dogs were *a*^*w*^ wolf sable, *a*^*t*^ tan point or *a* recessive black determined by their A locus (due to only wild type variant *k*^*y*^ being present on the K locus of these dogs). A maximum copy number of two allelic variants at the A locus was found in any of the Tamaskan Dogs and no *a*^*y*^ variant, indicating that no A locus anomalies were present as recently observed in a small number of other dog breeds [12]. The observed coat color phenotypes were in concordance with expected phenotypes for 26 of the Tamaskan Dogs, while 17 of the Tamaskan Dogs manifested more abundantly pheomelanic hairs in areas of head, legs and body on which the coat color pattern known as “domino” or “grizzle” is formed. This patterning, which is commonly observed in the two arctic breeds Alaskan Malamute and Siberian Husky, bears high phenotypic resemblance to previously characterized *E*^*G*^ domino in Afghan Hound and *E*^*G*^ grizzle in Saluki. The aforementioned have been suggested to be dependent on the A locus *a*^*t*^/*a*^*t*^ genotype for their manifestation [3]. Here, the domino pattern is observed independently from *E*^*G*^ on divergent breed backgrounds (Fig. 2). Domino phenotype encompasses pale facial markings with receded eumelanin line forming a widow’s peak in the forehead, and often also white markings expressed up the centerline of the face including reduced pigment in the centerline of the nose referred to as a dudley nose. The latter phenotypic feature (white markings and a dudley nose) is also common in recessive red dogs. Notably, two dogs expected to manifest solid black coat as a result of *a*/*a* genotype on their A locus also showed lightened body coat color with tan point like markings that were very profound in the newborn puppy, while the coat phenotype resembled wild sable or tan point in the adult recessive black dog (Fig. 2 and Fig. 3; u- x).

The 26 non-domino Tamaskan Dogs had one copy of the R301C variant in combination with either one copy of the *E* or *E*^*M*^ variant. Presence of two copies of the R301C variant (*N* = 4), or compound heterozygosity of *e*^*A*^ with *e* recessive red (*N* = 13), showed statistically significant association with domino phenotype (*P* = 2.37^− 12^) (Table 1). The phenotypic impact of the *e*^*A*^ allele - recessivity to wild type *E* and dominance to *e* - is further demonstrated in a litter of Tamaskan Dogs (Fig. 2). Thirty-nine additional Tamaskan Dogs without any copies of the *e*^*A*^ allele manifested a non-domino phenotype, suggesting that the *e*^*A*^ allele explains the presence of all domino phenotypes observed in Tamaskan Dog.

**Table 1:** E locus genotype results in Tamaskan Dog and their association with domino phenotype

Observed phenotype	2 copies of *e*^*A*^	1 copy of *e*^*A*^ with 1 copy of *e*^*1*^	1 copy of *e*^*A*^ with 1 copy of either *E*^*M*^ or *E*	Totals
domino	4	13	0	17
non-domino	0	0	26	26
Totals	4	13	26	33

### *e*^*A*^ allele of *MC1R* is associated with partial recessive red phenotypes in multiple breeds

After associating the *e*^*A*^ allele with a coat color phenotype within a single breed, we pursued characterization of the phenotypic impact of the *e*^*A*^ allele across different breeds and coat color genotypes. Examination of additional dog owner-provided photos revealed that the *e*^*A*^ allele is associated with apparent partial recessive red coat color patterning. The coat color phenotype was altered in all 70 dogs (including the 17 Tamaskan Dog study sample) with the *e*^*A*^ allele present in homozygous form (*N* = 35) or in heterozygous form paired with the recessive red *e*^*1*^ allele (*N* = 35). These phenotypes manifested in dogs with *e*^*A*^*/e*^*A*^ and *e*^*A*^*/e*^*1*^ genotypes as follows. All seven dogs with *e*^*A*^*/e*^*A*^ or *e*^*A*^*/e*^*1*^ and *K*^*B*^ (or alternatively the intermediate *k*^*br*^) on the K locus express a non-solid and non-striped eumelanin shade phenotype (Table 2, Table S2 and Fig. 3; a-g). In five out of seven dogs, three Cirneco dell’Etna’s and two Drevers (a breed in which the striped *k*^*br*^ brindle pattern is observed), the phenotype is clear fawn and virtually indistinguishable from recessive red *e*^*1*^/*e*^*1*^ (Table 2, Table S2 and Fig. 3; a-b and y). Of the remaining two *K*^*B*^ dogs, one Siberian Husky is wolf sable and one mixed breed dog is tan point (modified into saddle tan) (Table 2, Table S2, Fig. 3; e-f). Given that the five clear fawn dogs have *a*^*y*^/*a*^*y*^ genotype, the wolf sable dog has *a*^*w*^/*a*^*t*^ genotype and the mixed breed has *a*^*t*^/*a* genotype on the A locus, we conclude that these dogs express the coat color pattern of their A locus despite the presence of one copy of dominant variant on the K locus.

**Table 2:** Summary of the *e*^*A*^ phenotypes

E locus	K locus	A locus	Expected phenotype (in the absence of ***e***^***A***^***)***	Observed ***e***^***A***^ phenotype	Number of observed dogs
*e*^*A*^*/e*^*A*^ *or e*^*A*^/*e*^*1*^	*K*^*B*^/*	*a*^*y*^/−	black (or brindle)	fawn	5
	*K*^*B*^/*	*a*^*t*^/−	black (or brindle)	tan point/saddle tan	1
	*K*^*B*^/*	*a*^*w*^/−	black (or brindle)	wolf sable	1
*e*^*A*^*/e*^*A*^ *or e*^*A*^*/e*^*1*^	*k*^*y*^/*k*^*y*^	*a*^*y*^/−	fawn	cream/fawn domino	4
	*k*^*y*^/*k*^*y*^	*a*^*w*^/−	wolf sable	wolf sable domino	40
	*k*^*y*^/*k*^*y*^	*a*^*t*^/−	tan point	domino/pied/grizzle/red	12
	*k*^*y*^/*k*^*y*^	*a*/*a*	recessive black	wolf sable/tan point	7
*E*/*e*^*A*^	*any*	any	various	none	39
*E*^*M*^/*e*^*A*^	*any*	any	various	none	16
*E*^*G*^*/e*^*A*^	*any*	*any*	various	*no available phenotypes*	*no observed dogs*

Altogether all 63 dogs with *e*^*A*^*/e*^*1*^ or *e*^*A*^*/e*^*A*^ genotype expressing A locus manifested altered phenotype (Table 2 and Table S2). Of 49 out of 56 dogs with *e*^*A*^*/e*^*1*^ or *e*^*A*^*/e*^*A*^ genotype expressing A locus *a*^*y*^ fawn, *a*^*w*^ wolf sable, *a*^*t*^ tan point produced domino color patterning, but in all of the 56 dogs with *e*^*A*^ genotype resulted in increase of pheomelanin expression. One of the four *a*^*y*^ fawn dogs manifested domino patterning on eumelanin shaded fawn phenotype, whereas three dogs with *e*^*A*^*/e*^*1*^ or *e*^*A*^*/e*^*A*^ genotype combined with *a*^*y*^ fawn were phenotypically similar to recessive red *e/e* dogs..All of the 40 *a*^*w*^ wolf sample dogs had domino patterning. Typical domino patterning was also manifested in eight of the 12 *a*^*t*^ tan point dogs, while in four out of 12 *a*^*t*^ tan point dogs variation in the level of pheomelanin expression was observed.. One Drever homozygous for the *e*^*A*^ allele had no visible increase in its coat color pheomelanin expression; the dog expresses normal tan points, but also the white markings on the centerline of the face and a dudley nose. In contrast, almost no eumelanin pigment is present in two Hungarian Hounds with *e*^*A*^/*e*^*1*^ genotype manifesting rich red coat color with white markings on the centerline of the face and a dudley nose (Table 2, Table S2). Moreover, in one *a*^*t*^ tan point Beagle in which tan point coat color modifier Saddle Tan [20] is present, the ‘saddle’ consists of only a few eumelanic hairs and the dog manifests a dudley nose (Fig. 3; r). This resulting coat color phenotype in the Beagle breed is called as “pied”, and we now demonstrate it to be caused by *e*^*A*^ ancient red. In addition, all seven out of seven *a* recessive black dogs had pheomelanic markings despite of the loss-of-function *a* variantresulting in coat phenotypes resembling tan point or wolf sable (Table 2, Table S2, Fig. 3; h-i and u-w).

We observed no phenotype change in 52 dogs genotyped *E*^*M*^/*e*^*A*^ (*N* = 16) or *E*/*e*^*A*^ (*N* = 37) strongly suggesting an allelic hierarchy in which *e*^*A*^ is recessive to *E*^*M*^ and *E* and dominant to *e*, while further information on phenotypes produced by *E*^*G*^/*e*^*A*^ genotype remains to be collected (Table 2 and Table S2). In two Siberian Huskies with one copy of *e*^*A*^ and no other tested E alleles present the phenotype was altered to domino as if no wild type *E* was present. We did not have DNA availability to test for the presence of a rare *e*^*3*^ variant discovered in Huskies [1], but we hypothesize that the actual genotype of these dogs is *e*^*A*^/*e*^*3*^ based on the observed phenotype.

Taken together, phenotype data available in 15 different breeds consistently shows that *e*^*A*^ results in various increased pheomelanin pigment-containing phenotypes that we interpret to be partial recessive red coat colors. In dogs with *K*^*B*^ dominant black or *k*^*br*^ genotype, the K locus is masked and A locus is expressed instead, while in dogs expressing the A locus (in the absence of *K*^*B*^ variant) the ability to produce eumelanin is reduced resulting in coat color patterns known by the names “domino”, “grizzle” and “pied” depending on the breed background, but may also result in phenotypes indistinguishable from recessive red (cream), tan point or wolf sable.

## Discussion

Various *MC1R* gene polymorphisms have been documented and associated with pigment variation in human and domestic animals, and several coat color associated MC1R polymorphisms have already been identified in dogs. The goal of this study was to screen for the presence of an ancient R301C variant in today’s dog population and determine if it has any effect on coat color phenotypes putatively selected for during the early stages of domestication. The R301C variant of *MC1R* was first identified in over 10,000-year-old prehistoric dog DNA samples as a variant absent from wolf DNA samples [18]. Our study sample consisted of nearly 12,000 samples representing over 300 modern dog breeds and breed varieties which were genotyped for the presence of R301C and known coat color alleles as a part of a commercial genetic testing service (MyDogDNA™/Optimal Selection™). We confirm presence of the R301C variant in 35 dog breeds with a variant frequency of 1.5% in all dogs genotyped. A frequency of 50% or higher for R301C was observed in Spitz breeds (Alaskan Klee Kai, Northern Inuit Dog, Siberian Husky), and in Hound breeds (Drever, English Foxhound, Plott and Serbian Hound).

Phenotype analysis combined known coat color variant genotypes for *MC1R*, *CBD103* and *ASIP* genes with phenotype information from photos provided by the owners of the tested dogs. R301C was not found to be linked with any of the tested E locus variants (*E*^*M*^, *E*^*G*^ or *e*^*1*^). Phenotype analysis further suggests that the R301C variant is a novel reduced-function allele at the E locus, recessive to *E*^*M*^ and wild type *E*, but dominant to the *e* allele. Further support for genetic causality is provided by the association of the identical R301C mutation in *MC1R* with light coat color in two additional species; alpaca and Arabian camel [21, 22]. A similar cytosine to thymine mutation at the homologous base pair position (901) resulting in an S83F amino acid change is also associated with Chestnut color in horse [23]. The R301C mutation is located in the cytosolic C-terminal extension of the protein, shown to be functionally relevant for the cell surface expression of G protein-coupled receptors like *MC1R* [24, 25]. We demonstrate that the newly characterized allele, which we have termed *e*^*A*^, impacts phenotypes regulated by the K and A loci. We observed a loss of solid eumelanin shade in *K*^*B*^ dogs and receding eumelanin expression in color patterns produced by the A locus, and note that *e*^*A*^ behaves as a partially recessive red variant. The most recognizable *e*^*A*^ produced phenotype pattern in *a*^*w*^ wolf sable, *a*^*t*^ tan point and also in some *a*^*y*^ fawn dogs has been referred to as ‘Husky domino’ seen in Alaskan Malamute or Siberian Husky, but the molecular cause for this phenotype has remained unknown until now. Also, we now demonstrate that *e*^*A*^ is producing the coat color phenotype called “pied” in Beagle. For *e*^*A*^ phenotype to be expressed this variant allele of E locus needs to be present in homozygous form or in heterozygous form with *e* as the second allele. We observed two Siberian Huskies manifesting a domino phenotype while carrying one copy of *e*^*A*^ and no other tested E locus variant, which according to current conventions would be interpreted as wild type *E* being the second allele at the locus in these dogs. Although we did not have DNA sample availability to test for the presence of the recently discovered additional *e* alleles, *e*^*2*^ and especially *e*^*3*^ discovered in Huskies [1], we suggest that an *e*^*A*^/*e*^*3*^ genotype is the most likely explanation for the domino phenotype in these dogs.

We propose that the newly characterized reduced-function variant R301C is designated *e*^*A*^, where “*e”* is chosen for its *partial* recessive red identity and “A” is denoting “Ancient” that is recessive to *E* wild type and dominant to the e^1–3^ alleles. We propose an updated dominance hierarchy at the E locus; *E*^*M*^ > *E > e*^*A*^ > *e*^*1–3*^, while acknowledging that we could not identify any dog with the *E*^*G*^*/e*^*A*^ genotype and thus, the phenotype impact of this specific rare genotype combination remains unexplored. Interestingly, the previously identified domino variant *E*^*G*^ is almost exclusively observed in breeds in which *e*^*A*^ is not detected [6]. In this study, we found both *E*^*G*^ and *e*^*A*^ allele present in only one breed, Kritikos Lagonikos; a primitive hunting dog originating from the Greek island of Crete. Phenotype analysis of this rare breed might help to resolve the phenotypes presented by rare allele combinations, for which conclusions could not be made through this study.

Based on our phenotype analysis, common to *e*^*A*^ associated patterning is an characteristically increased expression of pheomelanin and decrease in the level of eumelanin expression, indicating that *e*^*A*^ represents a reduced-function variant of *MC1R* as postulated [18]. It is plausible that reduced-function of MC1R manifests as the partial recessive red phenotypes enabling some eumelanin to be expressed as observed by us, while other previously characterized recessive red alleles *e*^*1–3*^ represent loss-of-function variants of *MC1R* enabling expression of pheomelanin only when present in two copies. Consequently, *e*/*e* dogs always only express pheomelanin pigment regardless of variants present at the K locus and A locus, whereas the R301C variant *e*^*A*^ results in reduced MC1R function masking expression of the K locus in dominant black dogs while pigmentation phenotypes correlated with the expression pattern of the A locus. Logically on the other hand, increased pheomelanin expression is observed in dogs expressing the A locus due to the effects of *e*^*A*^. We propose that the *e*^*A*^ variant is a *partial* loss-of-function variant not previously known to be present in dogs, which furthermore provides novel insights into the relationship of variants at the E, K and A loci. The dominant allele of the K locus (a neutral antagonist of *MC1R* acting epistatic to the A locus when the *e*/*e* genotype is not present [13]) needs a *more* functional variant of the E locus for uniform eumelanin pigmentation than the level of functionality inherent to the *e*^*A*^ variant. In contrast, *ASIP,* an inverse agonist of *MC1R*, retains its function in the presence of the less functional *e*^*A*^ variant. Thus, it is suggested that in the presence of *e*^*A*^/*e*^*A*^ and *e*^*A*^/*e*^*1*^ genotypes, a reverse epistasis relationship between K and A locus occurs. Interestingly, the effect of *e*^*A*^ in genetically *a* recessive black dogs results in wild type or tan point-like patterning similar to *a*^*w*^ and *a*^*t*^ phenotypes. This allows us to postulate that also the *a* variant loss-of-function phenotype is dependent on the specifics of the *MC1R* variant type, where interaction with the *e*^*A*^ variant may be able to restore some of its function. Alternatively, this may be indicative of a potential non-causal role for the R96C variant in expression of the recessive black phenotype in dogs.

While partial recessive red phenotypes are produced by *e*^*A*^/*e*^*A*^ or *e*^*A*^/*e*^*1*^ genotypes, two copies of the reduced- function allele *e*^*A*^ could allow a bit more eumelanin to be expressed than when the *e*^*A*^ allele is present with a loss of function allele *e*^*1*^. We observed a Drever with *e*^*A*^/*e*^*A*^ genotype expressing normal tan points with only reduced pigment in the centerline of the head (white star) and nose (dudley nose), respectively. Outside of this study sample, we have further observed full domino pattern present in this dog’s offspring with *e*^*A*^/*e*^*1*^ genotype. The most depleted eumelanin expression was observed in two Hungarian Hounds with *e*^*A*^/*e*^*1*^ genotype and *a*^*t*^/*a*^*t*^ genotype for tan points in the A locus; these two dogs were essentially indistinguishable from recessive red individuals of the same breed. In addition, it should be noted that the domino pattern in all *a*^*y*^ fawn dogs is not clearly visible and their phenotype is similar to recessive red.

Substantial variation in pigment phenotypes in human populations is also explained by *MC1R* polymorphisms, where reduced-function variants are present in both Europeans and Asians. It is commonly believed that these variants represent adaptation to higher latitudes enabling sufficient synthesis of D-vitamin under lower solar radiation, while the population-genetic analysis suggests relaxed functional constraints out of Africa and South-Asia to explain the *MC1R* polymorphisms in human [26]. In dogs that rely on nutritional intake of D-vitamin and do not show seasonal variation in serum D-vitamin concentrations [27], *MC1R* polymorphisms are unlikely to associate with D-vitamin intake. A more plausible explanation for increase in frequency of a phenotype changing variant early in the domestication process is positive selection of novel traits applied by humans [15, 16]. Confirming *e*^*A*^ being the major variant in some old Spitz and Hound type dogs bred mostly for utility suggests that R301C may have once been a common variant in the domesticated dog population that has become eradicated in more modern breeds to enable manifestation of other coat color phenotype associated variants.

MC1R is not only central to determination of pigment phenotype. Besides its role in stimulation of eumelanin synthesis to protect skin from UV radiation and DNA damage, MC1R has a physiological role in vascular homeostasis and cell migration [28], erythroblast differentiation [29], prevention of cartilage degradation [30], and dopaminergic neuron survival [31]. MC1R signaling activates antioxidant, DNA repair and anti-inflammatory pathways [32–34]. MC1R genotype affects the probability of developing malignant melanoma [35], nonmelanoma skin cancer [35–37], risk for developing complicated sepsis after trauma [38] and development of Parkinson’s disease [31, 39, 40] in humans. Loss- or reduced-function variants in human *MC1R* have also been investigated in the response to pain, analgesia and anesthetics [41–44]. Moreover, in Standard Poodle the *e*^*1*^/*e*^*1reduced-function variants in human*^ genotype has been shown to prevent clinical signs of disease in dogs carrying causal variant causing Squamous Cell Carcinoma of the Digit (SCCD) [45]. Further work is needed to establish the molecular effect of the reduced-function variant R301C, and to understand its potential effects in dogs beyond determining coat color pigmentation.

In summary, genotype to phenotype correlation characterizes a novel allele of the E locus, caused by an old polymorphism in the *MC1R* gene associated with reduced eumelanin pigment that potentially represents one of the earliest mutations enriched by canine domestication still present in the dog population. This ancient E locus variant (MC1R p. R301C), which we have designated as *e*^*A*^, is recessive to *E*^*M*^ and *E* alleles of the E locus and dominant to the *e* allele. The genotypes *e*^*A*^/*e* and *e*^*A*^/*e*^A^ result in phenotypically reduced expression of eumelanin, and these genotypes exhibit partial epistasis over the A locus expression pattern and epistasis over the K locus.

## Conclusions

This study represents a large genotype screening effort of pet dogs, aiming to identify the presence of and understand the potential effect of one of the earliest mutations captured by canine domestication. It underscores the crucial role of dog owners in citizen science and more specifically in supporting studies aiming to elucidate the genetic background of trait phenotypes. The present discoveries could only have been made by comprehensive screening of coat color variants across a large number of breeds and individuals, in combination with the openness of dog owners to submit pictures of their dogs for research purposes. In conclusion, our findings explain the non-eumelanin coat color phenotypes observed in some dogs despite presence of the dominant black allele on the K locus, and identify that the same molecular cause explains the coat color phenotype commonly referred to as “domino” in Alaskan Malamute and other Spitz breeds, “grizzle” in Chihuahua, and “pied” in Beagle.

## Methods

### Study sample

The study sample (*N* = 11,750) consisted of non-invasive cheek swab samples collected by dog owners, and either blood or cheek swab samples collected at certified veterinary clinics in accordance with international standards for animal care and research as a part of voluntary submission of samples to commercial DNA testing. In addition, the dog owners provided consent for the use of their dog’s DNA information for research purposes. The samples were submitted for MyDogDNA / Optimal Selection analysis at Genoscoper Laboratories (Helsinki, Finland) and Wisdom Health (formerly Mars Veterinary) between April 3rd, 2015 and June 23th, 2020. Most of the tested dogs were from Finland (*N* = 5005, 42.6%) and the United States (*N* = 3192, 27.2%). The other major subgroups were formed by dogs from the Netherlands (*N* = 774, 6.6%), Denmark (*N* = 598, 5.1%), Austria (*N* = 413, 3.5%), France (*N* = 300, 2.6%), UK (*N* = 290, 2.5%),, Sweden (*N* = 164, 1.4%) and Australia (*N* = 121, 1.0%). Most of the tested dogs were from breeds recognized by Fédération Cynologique Internationale (FCI) or American Kennel Club (AKC), and the breed of the dog was reported by its owner with accompanying registration information. A few additional breeds not yet recognized by any major breed registry but with an established number of breed hobbyists, and mixed breed dogs, were also included in the study sample. Altogether, it amounted to 304 breeds and breed varieties, and 391 dogs representing the mixed breed population.

### Genotyping

Genotyping of coat color gene variants of *MC1R* [2–5], *CBD103* [13, 14] and *ASIP* [7–9] loci, and the R301C variant of the *MC1R* gene was carried out according to manufacturer-recommended standard protocols on a custom-designed Illumina Infinium technology bead chip ( [46, 47], Illumina, San Diego, Ca, USA). The genotyping quality control measures for this platform were previously described in [46, 47]. For the purposes of this study, the R301C variant assay findings were additionally validated with a second genetic technology by Sanger sequencing in representatives of the breed Finnish Lapphund and Cirneco dell’Etna on a ABI3730xl DNA Analyzer platform (Thermo Fisher Scientific, Waltham, MA, USA) at the Finnish Institute of Molecular Medicine (FIMM) Sequencing Unit as described earlier in [46]. Primers used for sequencing of the R301C locus were: 5- ACACTCACTATCCTGCTGGG − 3 (forward) and 5-TATTCCTTTCTCTGGCCCCA-3 (reverse).

### Phenotypic association

Coat color phenotype analysis utilized customer provided photos of dogs where the evaluator of the dog’s phenotype was blind to the genotype. The phenotypic impact of the R301C variant was evaluated by considering the genotypes for R301C in conjunction with genotypes at the interacting coat color loci *MC1R*, *ASIP* and *CBD103.*

### Statistical analyses

The statistical significance of differences in the distribution of *e*^*A*^ variant between observed domino and non-domino phenotypes inside one breed (Tamaskan Dog) was evaluated with the Freeman-Halton extension of Fisher’s Exact Probability test for a 2 × 3 table [48]. Dogs with one copy of *e*^*A*^ and an *E*^*M*^ variant were combined with dogs carrying *e*^*A*^ with an *E* variant due to only one individual present in the aforementioned category.

## Supplementary Information

**Additional file 1 Table S1.** Allele frequency of R301C variant in the study sample composition.

**Additional file 2 Table S2.** Phenotype analysis of 125 dogs with known genotype for *MC1R*, *CBD103* and *ASIP* loci.

## Data Availability

All relevant data is provided with the paper and its Supporting Information files.
